# Expedited transfer to emergency department vs. cardiac catheter laboratory in a cardiac arrest centre for non-ST-elevation out-of-hospital cardiac arrest: ARREST trial as-treated analysis

**DOI:** 10.1093/ehjacc/zuaf133

**Published:** 2025-10-22

**Authors:** Tiffany Patterson, Gavin D Perkins, Matthew Dodd, Alexander Perkins, Adam Mellett-Smith, Rachael T Fothergill, Tim Clayton, Richard Evans, Steven Robertson, Matthew Kwok, Karen Wilson, Benedict McDonaugh, Paul McCrone, Miles Dalby, Sam Firoozi, Iqbal Malik, Roby Rakhit, Ajay Jain, Philip MacCarthy, Jerry P Nolan, Simon R Redwood, the ARREST Trial Collaborators, Anthony Lampard, Desiree Papadopoulos, Johanna Hughes, Valentina Pendolino, Joanna Shaw, Clara Bannister, Amy Long, Justin Kearney, Gabriel Palti, Joanne Ritches-Price, Mark Whitbread, Divaka Perera, James Coutts, Brian Clapp, Bernard Prendergast, Douglas Chamberlain, Keith Muir, Tim Morris, Rod Stables, Joanne Nevett, Michael Connor, Garth Lane, Martin Brown, Mamas Mamas, Dawn Adamson, Nick Curzen, Lucy Blows, Mark De Belder, Maciej Marciniak, Nigel Stephens, Masood Khan, Edward Petzer, Oliver Spencer, Ian Webb, Patrick Roberts, Darryl Wood, Antonis Pavlidis, Therese Sidney, Rita Das, Sukhjinder Nijjer, Muhiddin Ozkor, Arvinder Kurbaan, Robert Bell, Darryl Wood, Gareth Rosser, Lauren Jerome, Megan Knight, Andrew Wragg, Jonathan Byrne

**Affiliations:** Cardiovascular Department, Guy’s and St Thomas’ NHS Foundation Trust, Westminster Bridge Road, London SE1 7EH, UK; Cardiovascular Department, Faculty of Life Sciences and Medicine, King’s College London, London, UK; Clinical Trials Unit, Warwick Medical School, University of Warwick, Coventry, UK; London School of Hygiene and Tropical Medicine Clinical Trials Unit, London, UK; London School of Hygiene and Tropical Medicine Clinical Trials Unit, London, UK; Clinical Audit and Research Unit, London Ambulance Service, London, UK; Clinical Trials Unit, Warwick Medical School, University of Warwick, Coventry, UK; Clinical Audit and Research Unit, London Ambulance Service, London, UK; Faculty of Health, Social Care and Education, Kingston University, London, UK; London School of Hygiene and Tropical Medicine Clinical Trials Unit, London, UK; London School of Hygiene and Tropical Medicine Clinical Trials Unit, London, UK; London School of Hygiene and Tropical Medicine Clinical Trials Unit, London, UK; London School of Hygiene and Tropical Medicine Clinical Trials Unit, London, UK; Cardiovascular Department, Guy’s and St Thomas’ NHS Foundation Trust, Westminster Bridge Road, London SE1 7EH, UK; Cardiovascular Department, Guy’s and St Thomas’ NHS Foundation Trust, Westminster Bridge Road, London SE1 7EH, UK; Cardiovascular Department, Faculty of Life Sciences and Medicine, King’s College London, London, UK; Institute for Lifecourse Development, University of Greenwich, London, UK; Department of Cardiology, Brompton and Harefield NHS Foundation Trust, London, UK; Department of Cardiology, St Georges Hospital, London, UK; Department of Cardiology, Imperial College Healthcare NHS Trust, London, UK; Department of Cardiology, Royal Free Hospital Foundation Trust, London, UK; Department of Cardiology, Barts Heart Centre, London, UK; Department of Cardiology, King’s College Hospital, London, UK; Department of Anaesthesia, Royal United Hospital, Bath, UK; Clinical Trials Unit, Warwick Medical School, University of Warwick, Coventry, UK; Cardiovascular Department, Guy’s and St Thomas’ NHS Foundation Trust, Westminster Bridge Road, London SE1 7EH, UK; Cardiovascular Department, Faculty of Life Sciences and Medicine, King’s College London, London, UK

**Keywords:** Cardiac Arrest Centre, Out-of-Hospital Cardiac Arrest

## Abstract

**Aims:**

The ARREST trial demonstrated that in adult patients, transfer to a cardiac catheter laboratory in a cardiac arrest centre (CAC) following resuscitated out-of-hospital cardiac arrest (OHCA) without ST-elevation did not reduce deaths at 30 days compared with delivery to the geographically closest emergency department (standard care). More than half of the CACs had a co-located emergency department to which patients were delivered as part of the standard care arm, which may have influenced outcomes.

**Aims:**

We performed a pre-specified as-treated analysis to determine if a CAC and the location patients were delivered to, either emergency department or cardiac catheter laboratory, reduced deaths.

**Methods and results:**

Patients (aged ≥18 years) with resuscitated OHCA without ST elevation who were enrolled in the ARREST trial were grouped according to the location they were to delivered to– either an emergency department with or without a co-located CAC or a cardiac catheter laboratory within a CAC—at one of 35 hospitals in London, UK—by London Ambulance Service irrespective of randomized allocation. The as-treated population was therefore analysed as one of three groups: 1) emergency department in a CAC, 2) direct to a cardiac catheter laboratory in a CAC, and 3) emergency department in a non-CAC. The primary outcome of the trial was all-cause mortality at 30 days. Secondary outcomes included all-cause mortality at 3 months and neurological outcome at discharge and 3 months. A pre-specified analysis adjusting for age, sex, initial shockable rhythm, witnessed cardiac arrest, bystander CPR, the time from cardiac arrest until ROSC, and location of cardiac arrest was performed in the as-treated groups. Between 15 January 2018 and 1 December 2022, a total of 862 participants were enrolled into the trial. Data for the primary outcome for this analysis were available in 818/862 (94.9%). Patients delivered to an ED in a CAC had fewer deaths at 30 days compared with the ED in a non-CAC group (83/182, 45.6% vs. 178/233, 76.4%; adjusted OR 0.43, 95% CI 0.24 to 0.76; *P* = 0.0039). Patients delivered to a cardiac catheter laboratory in a CAC also had fewer deaths compared with the ED in a non-CAC group, but there was no statistical difference (250/403, 62.0%: adjusted OR 0.72, 95% CI 0.44 to 1.18; *P* = 0.19). Survival with a favourable neurological outcome at hospital discharge occurred in 88/177 (49.7%) of the ED in a CAC group, 130/406 (32%) of the catheter laboratory in a CAC group, and 42/228 (18.4%) of the ED in a non-CAC group.

**Conclusion:**

In this as-treated analysis of the ARREST trial, in adult patients with resuscitated OHCA without ST-elevation, we observed a lower 30-day mortality and favourable neurological outcomes following delivery to an ED in a CAC and cardiac catheter laboratory in CAC, when compared with delivery to ED in a non-CAC.

## Graphical Abstract

**Graphical Abstract:**
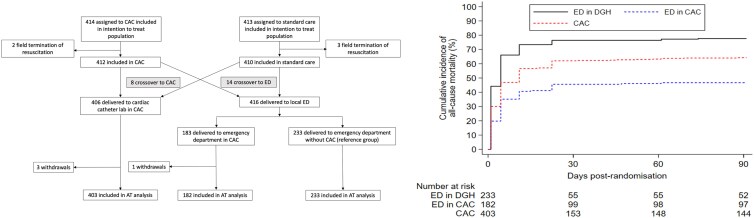
Flow diagram for the as-treated (left) and Kaplan Meier diagram (right). Abbreviations: CAC cardiac arrest centre, AT as-treated, ED emergency department

## Introduction

Delivery to a Cardiac Arrest Centre (CAC) following resuscitated out-of-hospital cardiac arrest (OHCA) of presumed cardiac cause may improve patient outcomes by concentrating services, increasing provider experience, and reducing the variability in healthcare infrastructure.^1–4^ The ARREST multi-centre randomised controlled trial demonstrated that in adult patients, transfer to a cardiac catheter laboratory in a cardiac arrest centre (CAC) following resuscitated OHCA without ST-elevation did not reduce deaths at 30 days compared with delivery to the nearest emergency department (ED) (standard of care).^5^ The secondary endpoints of all-cause mortality at 3 months and neurological outcome at discharge and 3 months also demonstrated no difference between the treatment arms.

The primary analysis of the ARREST trial was performed in the intention-to-treat (ITT) population according to allocated treatment arm.^6^ The ARREST trial was conducted by London Ambulance Service (LAS) NHS Trust across Greater London, United Kingdom, and included all 35 acute hospitals,^7^ of which were CACs. Four CACs had a co-located ED; these centres will have received patients delivered to the ED as part of the standard care arm if identified as the geographically closest hospital. CACs with co-located EDs would have had on-site 24/7 access to specialist cardiac facilities, in addition to a broad range of acute medical and surgical specialties serving the ED, capable of managing both cardiac and non-cardiac causes of arrest. This may have influenced patient outcomes.

We therefore performed an as-treated analysis of the primary endpoint of 30-day mortality and the secondary endpoints of 3-month mortality and neurological outcome at discharge and 3 months according to the location where patients were delivered.

## Methods

### Study design

The trial design, primary endpoint (30-day all-cause mortality) and secondary endpoints of 3-month all-cause mortality, and neurological outcomes (cerebral performance category and modified Rankin score) at discharge and 3 months have been reported previously in the ITT population.^5^ In brief, ARREST was an investigator-led, prospective, parallel, multicentre, open-label, randomised superiority trial. Adult patients with return of spontaneous circulation following OHCA without ST-elevation on their ECG were randomly assigned at the scene of their cardiac arrest to expedited delivery to the cardiac catheter laboratory at a CAC (intervention arm) or standard of care with delivery to the geographically closest ED.

**Figure 1:**
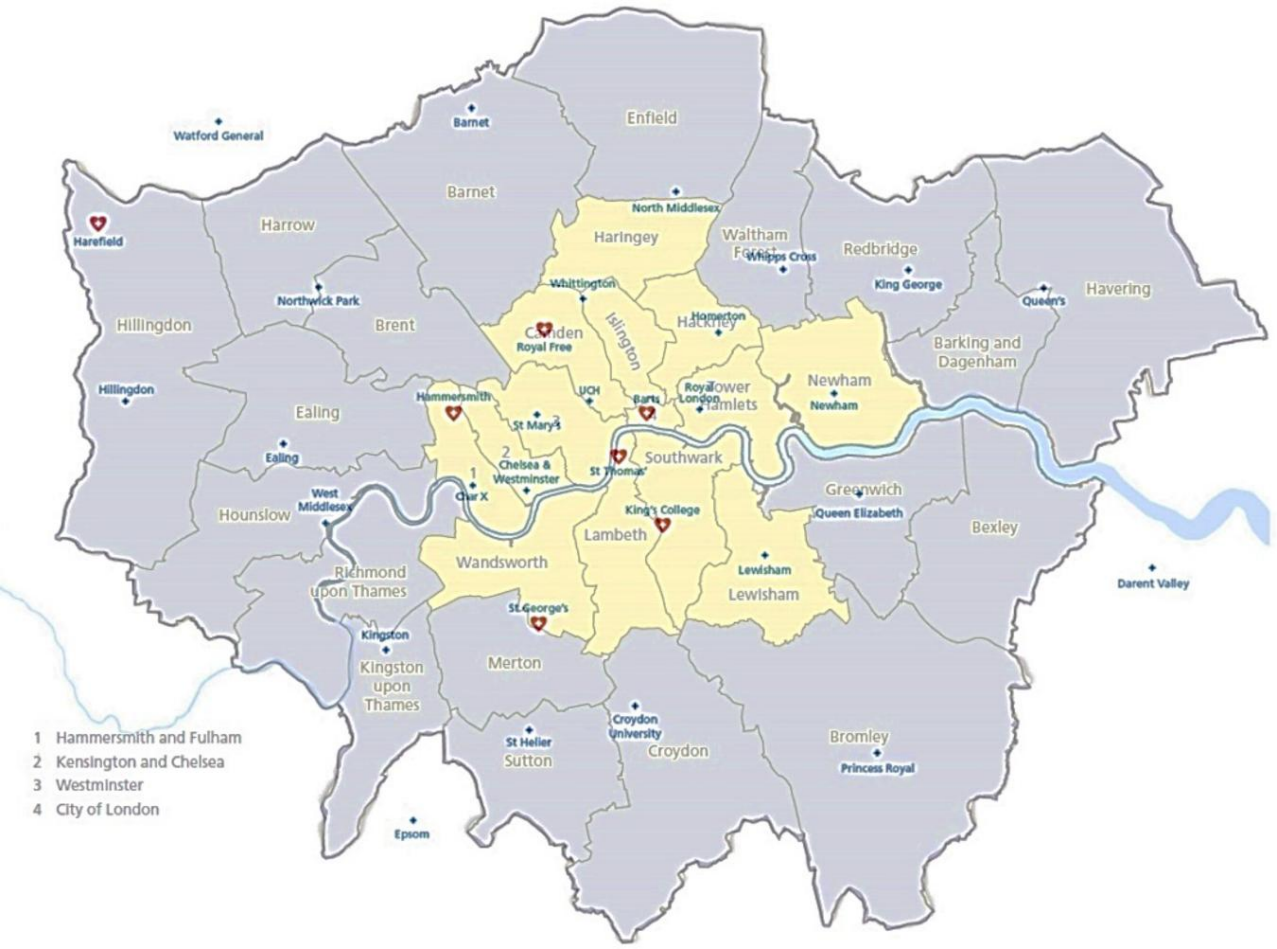
Map of London hospitals based on geographical location. Red hearts denote designated cardiac arrest centres. Yellow represents inner London, grey represents outer London. Hospitals labelled in blue.

The ARREST trial was coordinated by the London School of Hygiene and Tropical Medicine Clinical Trials Unit who also performed the statistical analyses, and was conducted 24 h a day, 7 days a week, by the LAS, the primary provider of prehospital emergency care across Greater London, and included all 35 centres (acute hospitals) capable of receiving resuscitated OHCA patients from LAS in London UK. Seven of these hospitals were CACs with emergency out-of-hours provision for interventional cardiology, cardiac surgery, and specialist intensive-care facilities. Four CACs had co-located EDs. The geographical spread of the 35 hospitals across London is shown in *Figure 1*. Details of the accessible facilities at the CACs and non-CACs have been previously provided.^5^

The trial was approved by the UK Health Research Authority and was prospectively registered prior to enrolment of the first patient (URL: https://www.isrctn.com; unique identifier: ISRCTN 96585404). The need for prior informed consent was waived because of the urgency of the intervention.^7^ Written informed consent was taken from the patient once the initial emergency had passed if they had regained capacity or from a personal or professional consultee. The Confidentiality Advisory Group granted permission (17/CAG/0151) to access patient-identifiable data under specific circumstances.

Of the 862 patients enrolled in the trial, 823 were included in the ITT population; 5 patients did not reach the hospital and therefore 818/862 (94.9%) were included in the present analysis. Full details of the inclusion and exclusion criteria have been previously provided;^5^ in brief, patients (aged ≥18 years) with return of spontaneous circulation following OHCA were deemed eligible for enrolment. Exclusion criteria were presumed non-cardiac cause, pregnancy, criteria for ST-elevation myocardial infarction on the post-resuscitation 12-lead electrocardiogram (ECG), presumed significant trauma/injury, or presence of a do-not attempt-resuscitation order.

### Procedures

The intervention arm comprised activation of the cardiac arrest pathway (24/7) with pre-alert of the receiving centre to the imminent arrival of a resuscitated cardiac arrest with strategic delivery to the catheter laboratory.^5,6,8^ Following delivery to the cardiac arrest centre, the patient was assessed by the on-call team (including but not limited to cardiologist and intensive care doctor). Due to the heterogeneity of possible diagnoses, the trial did not dictate in-hospital management which was left to physician discretion and expertise. CACs were fully equipped and had access to guideline-directed therapies including tracheal intubation and ventilation, haemodynamic support and monitoring, assessment of the underlying cause of arrest with on-site diagnostics, immediate coronary reperfusion and/or haemodynamic mechanical support devices and temperature control. The control arm comprised delivery to the geographically closest ED. Once the patient had been delivered to the assigned centre, they were considered as having completed allocated trial treatment. Both treatment arms mandated delayed neuroprognostication after 72 h prior to any withdrawal of life-sustaining treatment.^9^

### Randomization and data collection

Randomization was performed by LAS advanced paramedic practitioners based in the control room using a secure online randomization system. Following resuscitated OHCA, attending paramedics randomly assigned patients (1:1) using block permutation (sizes 4 and 6) without stratification to expedited transfer to a CAC or standard-of-care treatment (delivery to the closest ED). It was not deemed possible to mask clinicians, ambulance staff, or participants to the assigned group due to the radically different treatment groups. Clinical follow-up was performed up to 12 months.

### Outcomes

The primary endpoint of all-cause mortality at 30 days and the secondary endpoint of neurological (functional) outcomes at discharge and 3 months were assessed by the clinical teams, research nurses, or research paramedics. All-cause mortality at 6 and 12 months and health-care costs and cost-effectiveness will be reported separately. Survival with favourable neurological outcome was defined as having a modified Rankin Scale (mRS) score of 3 or less.^10^

### Statistical methods

Full details of statistical analyses and sample size calculation can be found in the statistical analysis plan, which has been previously published.^5^ Analysis of the primary endpoint was an unadjusted comparison of all-cause mortality at 30 days after randomization between those randomized to expedited transfer to a CAC or standard of care in the ITT population. The current pre-specified analysis was performed in the as-treated population according to the following definition where patients were considered as three groups based on the location they were delivered: 1) ED in a CAC, 2) direct to a cardiac catheter laboratory in a CAC, and 3) ED in a non-CAC. For this analysis, the main comparisons were with group 3: i.e. ED in a non-CAC (the standard care arm and reference group). Unadjusted risk ratios (RR) and odds ratios (OR) with corresponding 95% confidence intervals (CI) and *P*-values were calculated using generalized linear models for binomial outcomes with a log link and logit link function, respectively. For this as-treated analysis, a pre-specified analysis adjusting for the following variables was also performed: age, sex, initial shockable rhythm, witnessed cardiac arrest, bystander CPR, the time from cardiac arrest until ROSC, and location of cardiac arrest (at home or in public). Due to model convergence issues when estimating adjusted RRs, only adjusted ORs are reported. Multiple imputation by chained equations was used to impute missing values in these variables (20 iterations). Neurological status using the mRS as the primary neurological outcome measure was compared between the three groups with ordered logistic regression at discharge and 3 months. Statistical analyses were performed using Stata 18 (Stata Corp LLC, College Station, TX) and R version 4.3.0 (r-project.org).

## Results

**Figure 2:**
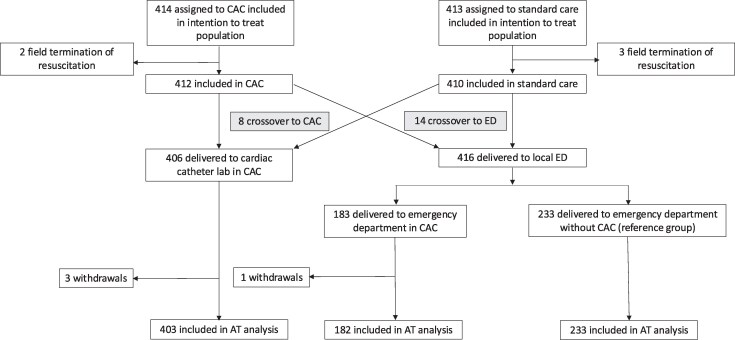
Flow diagram for the as-treated population. Abbreviations: CAC cardiac arrest centre, AT as-treated, ED emergency department.

Between 15 January 2018 and 1 December 2022, a total of 862 participants were enrolled and randomized by LAS. Among the 862 participants, data for the primary endpoint were available in 818/862 (94.9%) patients in the as-treated analysis. The final participant completed 12 month follow-up in November 2023. The as-treated population was separated into three groups: ED in a CAC: 182/818 (22.5%), direct to a cardiac catheter laboratory in a CAC: 403/818 (49.3%) and ED in a non-CAC 233/818 (28.5%) (*Figure 2*).

The baseline characteristics are provided in *Table 1*. The ED in a CAC group was on average younger with a higher proportion of male patients; they had a lower incidence of diabetes and chronic renal failure and were more likely to be non-smokers compared to the other two groups.

**Table 1:** Baseline characteristics of the as-treated population

Characteristic	Emergency department in CAC n = 182	Cardiac catheter laboratory in CAC n = 403	Emergency department in non-CAC n = 233
*Demographics*			
Age—years	61 (15.1)	64 (15.5)	65 (16.5)
Male sex	130/182 (71.4)	281/403 (69.7)	145/233 (62.2)
Female sex	52/182 (28.6)	122/403 (30.3)	88/233 (37.8)
*Ethnicity*			
Caucasian	104/182 (57.1)	218/403 (54.1)	123/233 (52.8)
Asian	31/182 (17.0)	68/403 (16.9)	39/233 (16.7)
Afro-Caribbean	11/182 (6.0)	20/403 (5.0)	15/233 (6.4)
Other	12/182 (6.6)	39/403 (9.7)	33/233 (14.2)
Not known	24/182 (13.2)	58/403 (14.4)	23/233 (9.9)
*Medical history*			
Diabetes	38/174 (21.8)	97/376 (25.8)	53/207 (25.6)
Hypertension	83/168 (49.4)	181/368 (49.2)	106/208 (51.0)
Smoking status			
Never smoked	54/182 (29.7)	93/403 (23.1)	30/233 (12.9)
Ex-smoker	26/182 (14.3)	48/403 (11.9)	28/233 (12.0)
Current smoker	27/182 (14.8)	40/403 (9.9)	29/233 (12.4)
Not known	75/182 (41.2)	222/403 (55.1)	146/233 (62.7)
Hypercholesterolemia	39/157 (24.8)	99/334 (29.6)	44/162 (27.2)
Peripheral vascular disease	6/168 (3.6)	12/353 (3.4)	7/183 (3.8)
Cerebrovascular disease	12/170 (7.1)	26/362 (7.2)	27/195 (13.8)
Chronic renal failure	9/171 (5.3)	33/365 (9.0)	22/197 (11.2)
Known ischaemic heart disease	27/169 (16.0)	83/354 (23.4)	35/189 (18.5)
Previous myocardial infarction	18/169 (10.7)	54/356 (15.2)	29/197 (14.7)
Previous percutaneous coronary intervention	18/166 (10.8)	45/352 (12.8)	16/189 (8.5)
Family history of heart disease	21/100 (21.0)	32/179 (17.9)	11/66 (16.7)
*Preceding symptoms prior to arrest*	61/129 (47.3)	118/262 (45.0)	85/134 (63.4)
Chest pain	18/61 (29.5)	30/118 (25.4)	24/85 (28.2)
Dizziness	9/61 (14.8)	9/118 (7.6)	22/85 (25.9)
Breathlessness	20/61 (32.8)	47/118 (39.8)	32/85 (37.6)
Palpitations	4/61 (6.6)	2/118 (1.7)	4/85 (4.7)
Other symptoms	29/61 (47.5)	59/118 (50.0)	47/85 (55.3)

Data are n (%), median (IQR), mean (SD), or n/N (%).

Abbreviations: CAC cardiac arrest centre.

Key pre-hospital events and baseline characteristics are provided in *Table 2*. The ED in a CAC group (94/182, 52%) and catheter laboratory in a CAC group (202/403, 50%) received fewer arrests from private residences compared with the ED in a non-CAC group (149/233, 64%). The ED in CAC group (44/182, 79%) and catheter laboratory in a CAC group (303/403, 75%) were more likely to be bystander witnessed arrests compared with ED in a non-CAC group (161/233, 69%). The ED in a CAC group (125/182, 69%) and catheter laboratory in a CAC group (226/403, 56%) had higher rates of initial shockable rhythm compared with ED in a non-CAC group (98/233, 42%). Both CAC groups were observed to receive fewer doses of adrenaline, had a shorter time to ROSC, shorter time to first defibrillation and more frequently received public access defibrillation when compared with the ED in a non-CAC group. The ambulance transit times were similar in the ED in a CAC (9 min IQR 7 to 13) and ED in a non-CAC group (9 min, interquartile range (IQR) 7 to 12); the longest transit time was seen in the catheter laboratory in a CAC group (17 min, IQR 11 to 24). Cardiac cause of the arrest was more frequent in reported in the ED in CAC group (126/161, 78.3%) and cardiac catheter laboratory in CAC group (261/342, 76.3%) when compared with the ED in a non-CAC group (118/167, 70.7%).

**Table 2:** Key events pre-hospital in the as-treated population

Pre-hospital events	Emergency department in CAC n = 182	Cardiac catheter laboratory in CAC n = 403	Emergency department in non-CAC n = 233
Location of arrest			
Private	94/182 (51.6)	202/403 (50.1)	149/233 (63.9)
Public	88/182 (48.4)	201/403 (49.9)	84/233 (36.1)
Witnessed arrest			
Bystander	144/182 (79.1)	303/403 (75.2)	161/233 (69.1)
London Ambulance Service	13/182 (7.1)	26/403 (6.5)	16/233 (6.9)
Not witnessed	25/182 (13.7)	74/403 (18.4)	56/233 (24.0)
Presenting cardiac rhythm			
AED non-shockable/asystole/PEA	57/182 (31.3)	176/403 (43.7)	135/233 (57.9)
AED shockable/VF/pulseless VT	125/182 (68.7)	226/403 (56.1)	98/233 (42.1)
Not known	0/182 (0.0)	1/403 (0.2)	0/233 (0.0)
Initial CPR attempt			
Bystander	142/182 (78.0)	286/403 (71.0)	168/233 (72.1)
London Ambulance Service	40/182 (22.0)	117/403 (29.0)	64/233 (27.5)
Not performed	0/182 (0.0)	0/403 (0.0)	1/233 (0.4)
Arrest to LAS CPR start			
Median (IQR) (mins)	10 (7 to 12)	9 (7 to 12)	9 (7 to 12)
Number of patients in analysis	n = 128	n = 274	n = 147
First defibrillation performed			
Public access defibrillator	37/182 (20.3)	48/403 (11.9)	16/233 (6.9)
London Ambulance Service	92/182 (50.5)	208/403 (51.6)	106/233 (45.5)
Not performed	48/182 (26.4)	135/403 (33.5)	105/233 (45.1)
Not known	5/182 (2.7)	12/403 (3.0)	6/233 (2.6)
Arrest to first defibrillation			
Number of patients in analysis	n = 104	n = 192	n = 93
Median (IQR) (mins)	9 (6 to 12)	10 (7 to 14)	11 (8 to 17)
Number of shocks delivered			
Number of patients in analysis	n = 106	n = 239	n = 119
Median (IQR)	2 (1 to 4)	2 (1 to 4)	2 (1 to 3)
Adrenaline administered	92/182 (50.5)	257/403 (63.8)	172/233 (73.8)
Adrenaline dose			
Number of patients in analysis	n = 91	n = 250	n = 168
Median (IQR) (mg)	2 (1 to 4)	2 (1 to 4)	3 (1 to 4)
Amiodarone administered	23/182 (12.6)	69/403 (17.1)	33/233 (14.2)
Amiodarone dose			
Number of patients in analysis	n = 21	n = 66	n = 29
Median (IQR) (mg)	300 (300 to 300)	300 (300 to 300)	300 (300 to 300)
Mechanical CPR	35/182 (19.2)	97/401 (24.2)	60/231 (26.0)
Arrest to ROSC			
Number of patients in analysis	n = 149	n = 301	n = 169
Median (IQR) (mins)	21 (14 to 32)	24 (15 to 33)	28 (18 to 36)
Arrest to hospital arrival			
Number of patients in analysis	n = 156	n = 322	n = 178
Median (IQR) (mins)	73 (59 to 94)	84 (68 to 104)	82 (68 to 100)
Ambulance Transfer Time			
Number of patients in analysis	n = 177	n = 400	n = 230
Median (IQR) (mins)	9 (7 to 13)	17 (11 to 24)	9 (7 to 12)
Post ROSC Electrocardiogram^a^			
ST-segment elevation	3/182 (1.6)	8/403 (2.0)	1/233 (0.4)
Bundle branch block	41/182 (22.5)	113/403 (28.0)	59/233 (25.3)
ST-segment depression and/or T wave changes	82/182 (45.1)	151/403 (37.5)	100/233 (42.9)
No acute changes	32/182 (17.6)	89/403 (22.1)	51/233 (21.9)
No electrocardiogram^b^	24/182 (13.2)	42/403 (10.4)	22/233 (9.4)

Data are n (%), median (IQR), mean (SD), or n/N (%)

Abbreviations: CAC cardiac arrest centre, AED automated external defibrillator, PEA pulseless electrical activity, VF ventricular fibrillation, VT ventricular tachycardia, CPR cardiopulmonary resuscitation, IQR interquartile range, ROSC return of spontaneous circulation, ECG electrocardiogram

^a^The electrocardiogram was reviewed independently after trial enrolment and randomization

^b^No electrocardiogram denotes no hard copy available after randomization

### Primary and secondary outcomes

The primary and secondary outcomes are provided in *Table 3*. 30-day all cause mortality was 83/182 (45.6%) in the ED in a CAC group (unadjusted RR for survival 0.60, 95% CI 0.50 to 0.71; *P* < 0.0001), 250/403 (62.0%) in the catheter laboratory in a CAC group (unadjusted RR for survival 0.81, 95% CI 0.73 to 0.90; *P* = 0.0001) and 178/233 (76.4%) in the ED in a non-CAC group.

**Table 3:** Data for the primary outcome and secondary outcomes

	ED in CAC	RR or OR	Adjusted OR	Cath lab CAC	RR or OR	Adjusted OR	ED non-CAC
Primary endpoint							
30-day mortality	83/182 (45.6)	RR 0.60 (0.50 to 0.71) *P* < 0.0001	0.43 (0.24 to 0.76) *P* = 0.0039	250/403 (62.0)	RR 0.81 (0.73 to 0.90) *P* = 0.0001	0.72 (0.44 to 1.18) *P* = 0.19	178/233 (76.4)
		OR 0.26 (0.17 to 0.39) *P* < 0.0001			OR 0.50 (0.35 to 0.73) *P* = 0.0002		
Secondary endpoints							
3-month mortality	85/182 (46.7)	RR 0.60 (0.51 to 0.71) *P* < 0.0001		259/403 (64.3)	RR 0.83 (0.75 to 0.91) *P* = 0.0002		181/233 (77.7)
Discharge mRS score	n = 176			n = 403			n = 228
0	56/176 (31.8)			68/403 (16.9)			23/228 (10.1)
1	20/176 (11.4)			24/403 (6.0)			8/228 (3.5)
2	8/176 (4.5)			21/403 (5.2)			5/228 (2.2)
3	3/176 (1.7)			14/403 (3.5)			6/228 (2.6)
4	1/176 (0.6)			10/403 (2.5)			1/228 (0.4)
5	5/176 (2.8)			16/403 (4.0)			7/228 (3.1)
6	83/176 (47.2)	OR 0.23 (0.15 to 0.35), *P* < 0.001	0.38 (0.23 to 0.62) *P* = 0.001	250/403 (62.0)	OR 0.48 (0.33 to 0.69), *P* < 0.001	0.72 (0.46 to 1.13) *P* = 0.152	178/228 (78.1)
3-month mRS score	n = 171			n = 391			n = 223
0	55/171 (32.2)			74/391 (18.9)			16/223 (7.2)
1	19/171 (11.1)			23/391 (5.9)			12/223 (5.4)
2	5/171 (2.9)			17/391 (4.3)			4/223 (1.8)
3	4/171 (2.3)			5/391 (1.3)			5/223 (2.2)
4	1/171 (0.6)			9/391 (2.3)			2/223 (0.9)
5	2/171 (1.2)			4/391 (1.0)			3/223 (1.3)
6	85/171 (49.7)	OR 0.21 (0.14 to 0.33) *P* = 0.001	0.35 (0.20 to 0.59) *P* < 0.001	259/391 (66.2)	OR 0.44 (0.30 to 0.65) *P* = 0.001	0.59 (0.37 to 0.96) *P* = 0.035	181/223 (81.2)
Discharge mRS score							
Favourable	87/176 (49.4)			127/403 (31.5)			42/228 (18.4)
Unfavourable	89/176 (50.6)			276/403 (68.5)			186/228 (81.6)
3-month mRS score							
Favourable	83/171 (48.5)			119/391 (30.4)			37/223 (16.6)
Unfavourable	88/171 (51.5)			272/391 (69.6)			186/223 (83.4)

CAC = Cardiac Arrest Centre; CI = Confidence Interval; ED = Emergency Department; OR = Odds Ratio; mRS = modified Rankins Score; RR = Risk Ratio; 1 = Reference Group.

Data are n/N (%), OR or RR with (95% CI) *Adjusted for the following prespecified variables: age, sex, public/private arrest, witnessed/unwitnessed arrest, bystander CPR, cardiac rhythm, and time from arrest to ROSC. Abbreviations: CAC cardiac arrest centre.

**Figure 3:**
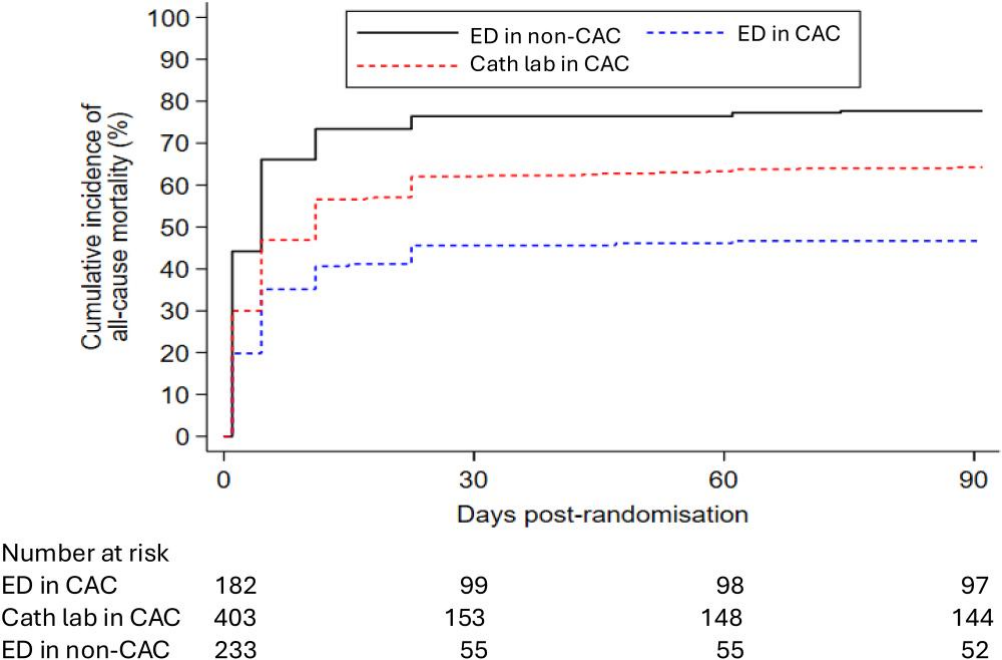
Kaplan-Meier for the as-treated analysis. Abbreviations: ED, emergency department; CAC, cardiac arrest centre.

The adjusted analysis for the primary endpoint of 30-day mortality was generally consistent with the results of the main analysis in terms of the direction of effect, though the evidence was less certain: ED in a CAC group (adjusted OR 0.43, 95% CI 0.24 to 0.76; *P* = 0.0039) and catheter laboratory in a CAC group (adjusted OR 0.72, 95% CI 0.44 to 1.18; *P* = 0.19) compared with the ED in a non-CAC group. There was also evidence of a difference in all-cause mortality at 3 months between the ED in a CAC group (unadjusted RR 0.60, 95% CI 0.51 to 0.71; *P* < 0.0001) and catheter laboratory in a CAC group (unadjusted RR 0.83, 95% CI 0.75 to 0.91; *P* = 0.0002) compared with ED in a non-CAC group. The Kaplan-Meier plot for 3-month mortality is provided in *Figure 3*.

Neurological outcomes at hospital discharge and at 3 months are provided in *Table 3*. There was a significant difference in mRS between the ED in a CAC group (OR 0.23, 95% CI 0.15 to 0.35; *P* < 0.0001) and the ED in a non-CAC group, this significance remained after co-variate adjustment. There was also a difference in mRS between the catheter laboratory in a CAC group (OR 0.47, 95% CI 0.32 to 0.68; *P* < 0.001) and ED in a non-CAC group, which did not reach statistical significance after co-variate adjustment. Survival with a favourable neurological outcome at hospital discharge occurred in 88/177 (49.7%) of the ED in a CAC group, 130/406 (32%) of the catheter laboratory in a CAC group and 42/228 (18.4%) of the ED in a non-CAC group.

## Discussion

In this as-treated analysis of the ARREST trial, in patients with resuscitated OHCA without ST-elevation, we observed a lower 30-day mortality following delivery to an ED in a CAC compared with ED in a non-CAC after adjustment for prespecified variables. The observed lower mortality following delivery to a cardiac catheter laboratory in a CAC compared with ED in non-CAC was not present after adjustment. We also observed higher rates of survival with favourable neurological outcome in patients delivered to an ED in a CAC and cardiac catheter laboratory in a CAC, but there was no difference after adjustment for prespecified variables.

Patients in both CAC groups, ED in a CAC and cardiac catheter laboratory in a CAC, were on average younger with shorter time to ROSC and first defibrillation, more likely to arrest in public and receive bystander CPR or public access defibrillation with higher incidence of an initial shockable rhythm; however, these factors were all adjusted for in the prespecified adjusted analysis. There were also important differences in populations of patients delivered to an ED in a CAC compared with an ED in a non-CAC, for example, in central London, the nearest ED is more likely to be a CAC, with a larger visiting population (*Figure 1*). In outer London, there are more residential areas, and the nearest ED is less likely to be a CAC. Ambulance transit time also differed between the groups, with transit time to the nearest ED nearly half that of delivery to a cardiac catheter laboratory in a CAC.

In the ITT analysis, the vast majority of patients were delivered to their assigned destination (406 to cardiac catheter laboratory and 416 to local ED with minimal crossover); the challenge arose when the local ED was CAC capable. In other words, when a patient had a cardiac arrest near an ED with a co-located CAC and was randomized to standard of care. In such circumstances it was not ethical to divert the patient to a non-CAC capable hospital, as standard of care would have been ED in a CAC. Such implementation issues cannot be easily overcome, and similar ethical issues likely arise in most major cities with a local population. The as-treated analysis was therefore conducted to understand how this may have influenced the trial outcome.

A large systematic review of 36 studies involving 147 943 patients treated in a CAC compared with a non-CAC observed improved survival with favourable neurological outcome in patients delivered to a CAC; however, the effect size reduced as the definition of CACs broadened.^11^ All designated CACs in the ARREST trial adhered to minimum recommendations for emergency out-of-hours provision for interventional cardiology, cardiac surgery, specialist intensive-care facilities, access to diagnostic imaging, and neuro-prognostication. However, three of the seven designated CACs were specialist cardiac units without an ED and hence did not adhere to the most recent guideline recommendations for multi-disciplinary infrastructure.^12^ The results of this current analysis suggest that delivery to a CAC may provide a survival benefit, with the strongest signal observed in those delivered to an ED in a CAC. Patients resuscitated from cardiac arrest without ST-elevation are a heterogeneous cohort. In this as-treated analysis, confirmed rates of cardiac cause of arrest were lower in the ED in a non-CAC group compared with the CAC groups (70% vs. 76% and 78%). Although a non-cardiac cause is unlikely to fully explain the difference in survival observed between the groups, it is possible that in cases where the preceding cause is unknown, delivery to an ED could provide a more multidisciplinary approach to diagnosis; ED co-location with a CAC has the added benefit of access to specialist cardiac care as required.

In their large systematic review, Yeo et al did not observe evidence of survival difference when bypassing centres to reach a CAC.^11^ Taken in the context of this current work, patients who were delivered to an ED in a CAC were taken there because it was the geographically closest hospital; whereas transit times direct to cardiac catheter laboratory in a CAC were double that of delivery to the nearest ED, which may have diluted the beneficial effect of the CAC. This could suggest that delivery to a CAC should aim to be within a certain time frame.

Another large systematic review of 22 studies involving 46 164 patients did not demonstrate evidence for difference in survival with favourable neurological outcome from treatment in a CAC after adjusted analysis, and the observational data are overall conflicting.^13^ The ARREST multicentre randomized trial did not demonstrate a reduction in all-cause deaths at 30 days in patients transferred to a cardiac catheter laboratory in a CAC compared with the geographically closest ED following resuscitated OHCA without ST-elevation. Prior to this, guidance from both the International Liaison Committee on Resuscitation (ILCOR) and the European Society of Cardiology (ESC) had recommended transfer of all resuscitated patients with non-traumatic OHCA to a CAC but with a low certainty of evidence, based on previously mentioned observational studies.^12,14^ Following ARREST, urgent questions still remain as to how best to implement pre-hospital systems of care for OHCA patients and how best to organize hospitals to ensure the optimal care delivery and outcomes.

This current analysis provides new insights into the association between the location patients were delivered (cardiac catheter laboratory or ED), additional expertise within the centres (multi-disciplinary infrastructure), and survival with favourable neurological outcome. In the post-arrest patient, in the absence of ST-elevation, it is possible that delivery to an ED in a CAC facilitates a multi-disciplinary approach to diagnosis and treatment; direct access to cardiac facilities if required but also early tracheal intubation, ventilation, and haemodynamic stabilization in addition to treatment for potential non-cardiac causes. More patients in the cardiac catheter laboratory in a CAC group were identified as being in cardiogenic shock; it is unclear if this was attributable to a lower threshold for diagnosis, increased access to mechanical support therapies, or longer time to reach the intensive care unit as the patient’s journey would have included a period in the cardiac catheter laboratory. Our data suggest that delivery to either an ED in a CAC or cardiac catheter laboratory in a CAC may provide a survival benefit over ED in a non-CAC, with stronger evidence in the ED in a CAC group which would be in keeping with previous trials of immediate vs. delayed coronary angiography in patients with non-ST elevation cardiac arrest.^15,16^

The decision to deliver the post arrest patient to a CAC in the absence of ST-elevation is a challenging one. Decision making should be tailored to the individual patient characteristics including haemodynamic instability and need for prompt in-hospital interventions; potential uncertainty of the cause of the arrest; geographical location of the arrest and therefore the ambulance transit time to the nearest ED vs. CAC; and if the ED has a co-located CAC with access to specialist cardiac care. Future randomized controlled trials of CAC conveyance may focus on pre-hospital selection criteria to better identify a cardiac cause of arrest and minimal neurological injury.^17^ In ARREST, absence of a clear non-cardiac cause was ‘presumed cardiac’ and there is potential to better identify underlying cardiac causes or acute ischaemia from the clinical history and by using Utstein criteria.^18,19^

The main limitation of this as-treated analysis is that the three groups were non-randomized which introduces selection bias and confounding factors that cannot be adjusted for. The ability to derive absolute conclusions from this data is limited and our findings are exploratory and hypothesis generating.

## Conclusions

In this as-treated analysis of the ARREST trial, in adult patients with resuscitated OHCA without ST-elevation, we observed a lower 30-day mortality and favourable neurological outcomes following delivery to an ED in a CAC and cardiac catheter laboratory in CAC, when compared with delivery to ED in a non-CAC.

## Data Availability

Individual patient data collected from the study, after de-identification and removal of any data not able to be shared under our regulatory agreements, will be made available to other researchers upon application to the ARREST trial: ARREST@lshtm.ac.uk.
